# Inflammatory demyelination alters subcortical visual circuits

**DOI:** 10.1186/s12974-017-0936-0

**Published:** 2017-08-18

**Authors:** Sheila Espírito Santo Araújo, Henrique Rocha Mendonça, Natalie A. Wheeler, Paula Campello-Costa, Kimberle M. Jacobs, Flávia C. A. Gomes, Michael A. Fox, Babette Fuss

**Affiliations:** 1 Department of Anatomy and Neurobiology, Virginia Commonwealth University School of Medicine, Richmond, VA USA; 2 Instituto de Biofísica Carlos Chagas Filho, Universidade Federal do Rio de Janeiro, Rio de Janeiro, Brazil; 3 Instituto de Biologia, Programa de Neurociências, Universidade Federal Fluminense, Niterói, Brazil; 4 Instituto de Ciências Biomédicas, Universidade Federal do Rio de Janeiro, Rio de Janeiro, Brazil; 5 Developmental and Translational Neurobiology Center, Virginia Tech Carilion Research Institute, Roanoke, VA USA

**Keywords:** Inflammation, Demyelination, Cuprizone, Multiple sclerosis, Glia, Synaptopathy, Visual system, Dorsal lateral geniculate nucleus (dLGN)

## Abstract

**Background:**

Multiple sclerosis (MS) is an inflammatory demyelinating disease classically associated with axonal damage and loss; more recently, however, synaptic changes have been recognized as additional contributing factors. An anatomical area commonly affected in MS is the visual pathway; yet, changes other than those associated with inflammatory demyelination of the optic nerve, i.e., optic neuritis, have not been described in detail.

**Methods:**

Adult mice were subjected to a diet containing cuprizone to mimic certain aspects of inflammatory demyelination as seen in MS. Demyelination and inflammation were assessed by real-time polymerase chain reaction and immunohistochemistry. Synaptic changes associated with inflammatory demyelination in the dorsal lateral geniculate nucleus (dLGN) were determined by immunohistochemistry, Western blot analysis, and electrophysiological field potential recordings.

**Results:**

In the cuprizone model, demyelination was observed in retinorecipient regions of the subcortical visual system, in particular the dLGN, where it was found accompanied by microglia activation and astrogliosis. In contrast, anterior parts of the pathway, i.e., the optic nerve and tract, appeared largely unaffected. Under the inflammatory demyelinating conditions, as seen in the dLGN of cuprizone-treated mice, there was an overall decrease in excitatory synaptic inputs from retinal ganglion cells. At the same time, the number of synaptic complexes arising from gamma-aminobutyric acid (GABA)-generating inhibitory neurons was found increased, as were the synapses that contain the *N*-methyl-d-aspartate receptor (NMDAR) subunit GluN2B and converge onto inhibitory neurons. These synaptic changes were functionally found associated with a shift toward an overall increase in network inhibition.

**Conclusions:**

Using the cuprizone model of inflammatory demyelination, our data reveal a novel form of synaptic (mal)adaption in the CNS that is characterized by a shift of the excitation/inhibition balance toward inhibitory network activity associated with an increase in GABAergic inhibitory synapses and a possible increase in excitatory input onto inhibitory interneurons. In addition, our data recognize the cuprizone model as a suitable tool in which to assess the effects of inflammatory demyelination on subcortical retinorecipient regions of the visual system, such as the dLGN, in the absence of overt optic neuritis.

## Background

Multiple sclerosis (MS) has long been considered a chronic inflammatory and demyelinating disease of the CNS that is ultimately associated with axonal degeneration and neuronal loss [1]. More recently, however, synaptic changes, possibly reflecting forms of maladaptive plasticity, have been implicated in contributing to the pathophysiology of the disease, even at early stages of disease progression when such changes may still be reversible [2, 3]. In this context, studies using experimental autoimmune encephalomyelitis (EAE) as a model suggest that synaptic changes in MS may include a shift toward increased excitation, which may be caused, at least in part, by T cell-initiated adaptive immune responses [2–7].

In the healthy CNS, appropriate synaptic functions and neural circuitries are regulated by resident micro- and astroglia cells, which contribute to synapse formation, elimination and plasticity, as well as neurotransmission [8]. Activation of CNS-resident glia cells under neuroinflammatory conditions, on the other hand, is thought to cause adaptive synaptic alterations ultimately leading to the malfunction of neural circuits [2, 6, 8, 9]. Synchronization of impulse traffic within neural circuits is likely also regulated by the extent of myelination [10]. Consistent with this point of view, demyelination has been implicated in causing delayed spike arrival timing as well as impaired activity coincidence and synaptic efficacy [11]. In MS, it is quite probable that maladaptive synaptic changes result from a combination of both neuroinflammation and demyelination, whereby the contribution of each of the two factors may depend on the affected brain area and/or the stage of the disease.

A common clinical manifestation in MS is visual dysfunction, which is often caused by inflammatory demyelination of the optic nerve, a condition known as optic neuritis [12]. However, not all patients with visual impairments exhibit optic neuritis, suggesting that neuroinflammation and demyelination of visual centers located within the brain may critically contribute to visual dysfunction in MS [13]. Likewise, in a common animal model of inflammatory demyelination, the cuprizone model, white matter areas and the cortex have been found affected while the optic nerve and tract are largely spared [14–16]. Thus, we used the cuprizone model to assess pathological changes, including potential synaptic alterations that are associated with inflammatory demyelination in the visual thalamus, an essential relay nucleus necessary for processing light-derived signals from the retina and transmitting those signals to the primary visual cortex [17]. Of note, inflammatory demyelination in deep gray matter including the thalamus has been reported to occur frequently in MS [18, 19].

## Methods

### Animals

Six-week-old C57BL/6 male mice (The Jackson Laboratory) were fed with chow containing 0.2% cuprizone (Sigma-Aldrich) or control chow for 3 or 5 weeks. All animal studies were approved by the Institutional Animal Care and Use Committee at the Virginia Commonwealth University.

### Antibodies

Anti-MBP (Aves Labs), anti-GFAP (DAKO), anti-F4/80 (clone Cl:A3-1; AbD Serotec), anti-PSD-95 (Abcam), anti-vGlut2 (Abcam), anti-GAD67 (Abcam), and Alexa488- and/or Alexa594-conjugated secondary antibodies (Molecular Probes) were used for immunohistochemistry. Anti-GluN2B (Synaptic Systems) antibodies were used for Western blot analysis and immunohistochemistry. Anti-GAPDH (EMD Millipore) and horseradish peroxidase (HRP)-labeled secondary antibodies (Vector Laboratories) were used for Western blot analysis.

### RNA isolation and real-time RT-qPCR analysis

Mice were deeply anesthetized using Avertin (2,2,2-Tribromoethanol, Sigma-Aldrich), and the brains were dissected into ice-cold PBS. Three hundred-micrometer coronal brain sections were prepared using a McIlwain tissue chopper (Ted Pella, Inc.), and the tissues, corpus callosum (CC) and dorsal lateral geniculate nucleus (dLGN), were dissected manually using small scalpel blades. The optic nerves were collected after removal of the brain. All tissue samples were flash frozen in liquid nitrogen or directly used for RNA isolation. RNA isolation and real-time RT-qPCR analysis was performed as described previously [20] using the following gene-specific primer pairs: *Mbp*: forward (5′-CTTGGCCACAGCAAGTACCATGGACC-3′) and reverse (5′-TTGTACATGTGGCACAGCCCGGGAC-3′); *Plp*1: forward (5′-CCACACTAGTTTCCCTGCTCACCT-3′) and reverse (5′-GGTGCCTCGGCCCATGAGTT-3′); *Il1b*: forward (5′-TGAAGAAGAGCCCATCCTCTGTGA-3′) and reverse (5′-GGTCCGACAGCACGAGGCTT 3′); *Tnf*: forward (5′-GCCCACGTCGTAGCAAACCACC-3′) and reverse (5′-CCCATCGGCTGGCACCACTA-3′); *Nos2*: forward (5′-TCCAGAATCCCTGGACAAGCTGC-3′) and reverse (5′-TGCAAGTGAAATCCGATGTGGCCT-3′); *Ptgs2*: forward: (5′-TTGCTGGCCGGGTTGCTGG-3′) and reverse: (5′-CAGGGAGAAGCGTTTGCGGT-3′); *Grin2a*: forward: (5’CGCATCCATGGCTTGGTGTTT-3′) and reverse (5′-TGTCGGATCCTTGTCAGCCAT-3′); *Grin2b*: forward (5′-ATGATGCCTTGCTCTCCCTG-3′) and reverse (5′-ATGCCATAGCCCGTAGAAGC-3′); *Pgk1* (reference gene): forward: (5′-ATGCAAAGACTGGCCAAGCTAC-3′) and reverse: (50-AGCCACAGCCTCAGCATATTTC-30). Relative expression levels were determined using the∆∆CT method [21].

### Immunohistochemistry

Mice were transcardially perfused with saline (0.9% NaCl) followed by 4% paraformaldehyde in 0.1 M phosphate buffer (pH 7.4). Brains were removed, post-fixed for 24 h, and coronal vibratome sections were collected at 40 μm (Leica VS1000S). The sections were permeabilized and blocked in phosphate-buffered saline (PBS) containing 3% bovine serum albumin (Sigma-Aldrich), 5% normal goat serum (NGS; Invitrogen), and 0.3% Triton X-100 and then incubated with primary (overnight at 4 °C) and secondary antibodies (2 h at RT). Primary and secondary antibodies were used at the following dilutions: chicken anti-MBP (1:500); rabbit anti-GFAP (1:500); rat anti-F4/80 (1:100); rabbit anti-PSD-95 (1:100); mouse anti-vGlut2 (1:400); rabbit anti-GluN2B (1:50); mouse anti-GAD67(1:500); Alexa Fluor 594 (goat anti-mouse IgG, 1:1000; goat anti-rabbit IgG, 1:1000); Alexa Fluor 488 (goat anti-chicken IgY, 1:500; rabbit anti-goat IgG, 1:300; goat anti-rabbit IgG, 1:400; goat anti-mouse IgG, 1:400). Sections were mounted using aqueous mounting medium (DAKO), and images were collected using a Leica TCS SPE confocal laser scanning microscope.

Quantification of immunohistochemical data was performed using ImageJ [22] by determining immunostained densities and the number of immuno-positive cells. Two fields per slice and two slices per animal were analyzed from four control and five cuprizone-treated mice. Synaptic puncta analysis was performed as described previously [23] using 20 serial optical sections captured at 0.71 μm intervals (Leica TCS SPE).

### Serial block face scanning electron microscopy

Mice were transcardially perfused sequentially with PBS and 4% paraformaldehyde/2% glutaradehyde in 0.1 M cacodylate buffer. Brains were immediately removed and vibratomed (300-μm coronal sections), and dLGN tissues were dissected from 300-μm coronal vibratome sections. Tissues were then stained, embedded, sectioned, and imaged by Renovo Neural Inc. (Cleveland, OH). Images were acquired at a resolution of 5 nm/pixel and image sets included > 200 serial sections (with each section representing 75 nm in the *z* axis). Serial block face scanning electron microscopy (SBFSEM) data sets were 40 μm × 40 μm × 12–20 μm. Retinal terminals and axons were identified and traced in TrakEM2 as described previously [24].

### Western blot analysis

Mice were deeply anesthetized using Avertin (2,2,2-Tribromoethanol, Sigma-Aldrich), and brains were dissected into ice-cold PBS. Three hundred-micrometer coronal brain sections were then prepared using a McIlwain tissue chopper (Ted Pella, Inc.), and dLGN tissue samples were obtained by manual dissection using small scalpel blades. The tissue samples were flash frozen in liquid nitrogen or directly homogenized in lysis buffer (150 mM NaCl, 10 mM KCl, 20 mM HEPES, pH 7.0, 1 mM MgCl_2_, 20% glycerol, and 1% Triton X-100, including the complete protease and phosphatase inhibitor cocktail (Thermo Fisher Scientific)). Twelve micrograms of protein per sample were separated by gel electrophoresis. After transfer and incubation with primary antibodies, HRP-conjugated secondary antibodies in combination with the ECL Prime Western blotting detection reagent (GE Healthcare Life Sciences) were used. Chemiluminescent signals were detected by exposure to photographic film (Kodak BioMax MR) and quantified by densitometry (Scion Image; Scion Corporation).

### Field potential recordings

Mice were transcardially perfused with ice-cold high-sucrose artificial cerebrospinal fluid (aCSF; in mM—2.5 KCl, 10 MgSO_2_, 0.5 CaCl_2_, 1.25 NaH_2_PO_4_, 26 NaHCO_3_, 11 glucose, and 234 sucrose; pH 7.4 when saturated with 95% O_2_–5% CO_2_). Parasagittal brain slices containing the optic tract reaching the dLGN were obtained as described by Chen and Regehr, 2000 [25]. Briefly, the brain hemispheres were separated by a 10° angled cut relative to the medial line. The intact hemisphere was attached to a 25° angled platform to prepare 300 μm thick vibratome slices. Using this procedure, one to two slices containing the OT reaching the dLGN could be collected from each brain. The slices were allowed to recover in oxygenated aCSF (in mM: 126 NaCl, 3.0 KCl, 2.0 MgCl_2_, 2.0 CaCl_2_, 1.25 NaH_2_PO_4_, 10 glucose, and 26 NaHCO_3_) at 34 °C for 1 h and at room temperature thereafter until placed in the recording chamber. Field potential recordings were made in slices placed in an interface chamber (Harvard Apparatus) with an Axoclamp2B amplifier and Cygnus ER1 for an overall gain of 1000X and digitized with a Digidata 1322A and pClamp software (all except Cygnus from Molecular Devices). During the recordings, the slices were maintained at 34 °C and continuously perfused with oxygenated aCSF. The tungsten stimulation and recording electrodes were positioned on the optic tract and dLGN, respectively, separated by a minimum distance of 100 μm. In order to assess the GluN2B component of the retinogeniculate field potential, baseline recordings were made in normal aCSF every 30 s for 5 min. Thereafter, Ro25-6981 (1 mM, Tocris Bioscience), a selective activity-dependent blocker of NMDA receptors containing the GluN2B subunit, was added to the perfusing aCSF, which likely required 5 min to reach the chamber. After 10 min, the recordings were made in the presence of the drug every 30 s for 5 min. In each slice, the peak amplitude of the synaptic-dependent field negativity post-drug was normalized using pre-drug values, whereby the second of two evoked negative field potential peaks was identified as the synaptic-dependent field potential by comparing recordings in normal aCSF to those in aCSF containing the competitive NMDA receptor antagonist APV (50 μM, Tocris Bioscience) and the competitive non-NMDA glutamate receptor antagonist DNQX (20 μM, Tocris Bioscience).

### Statistical analysis

For statistical analysis, the GraphPad Prism Software (GraphPad Software) was used. In the case data were compared with a normalized control value (1 or 100%), one-sample *t* tests were used [26, 27]. In the case of comparing two groups of data composed of variable samples, unpaired two-tailed Student’s *t* tests [28] were used. Normal distribution of the data was confirmed using Shapiro-Wilk and Kolmogorov-Smirnov normality tests. Sample size (*n*) refers to the number of animals and is indicated in each of the figure legends. Differences were considered significant for *p ≤* 0.05.

## Results

### In the cuprizone model, demyelination is apparent in visual centers located within the brain but not the optic nerve and tract

**Fig. 1:**
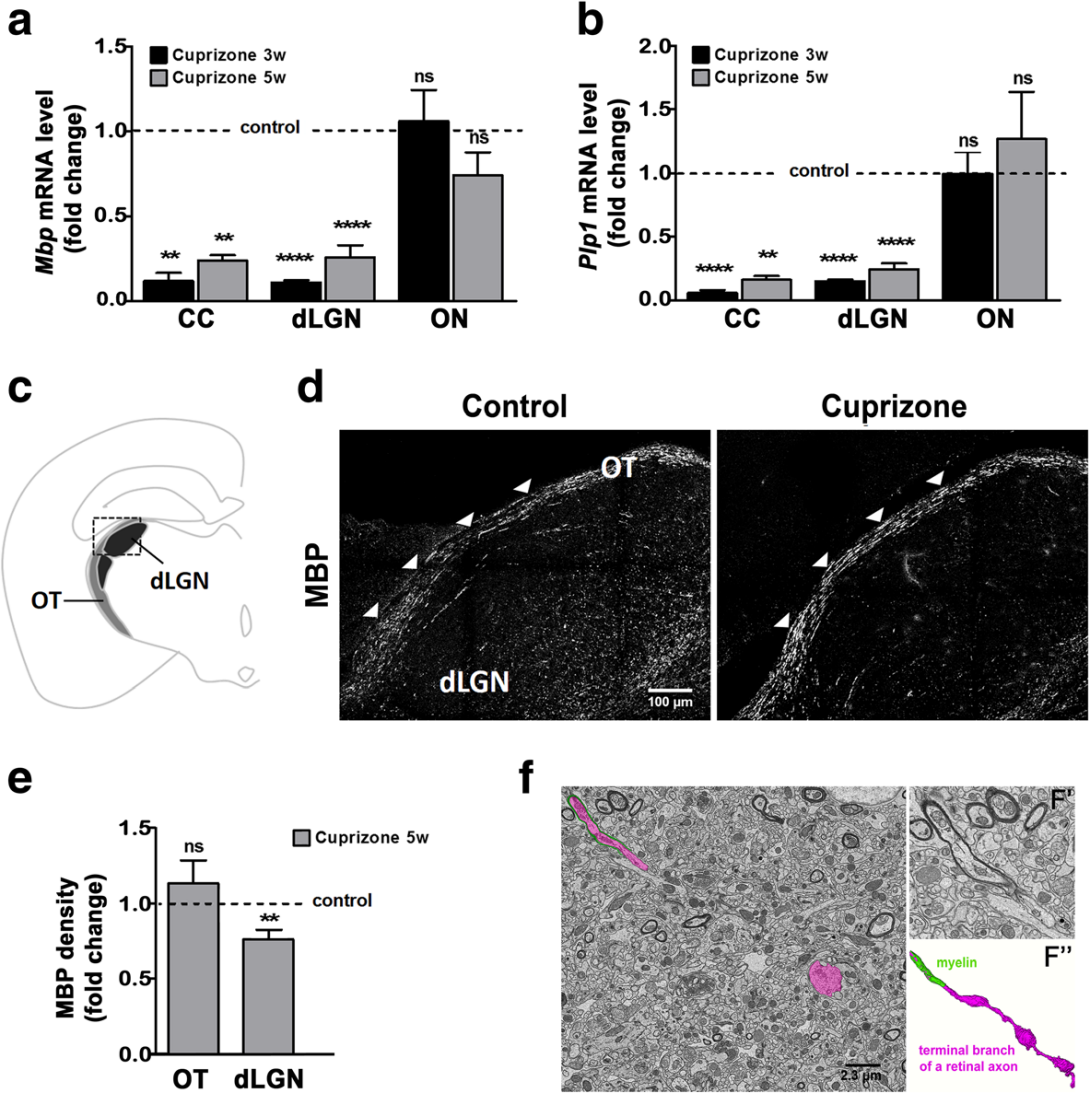
Cuprizone treatment leads to demyelination in the corpus callosum (CC) and dorsal lateral geniculate nucleus (dLGN), but not the retinogeniculate pathway, i.e., optic nerve (ON) and optic tract (OT). **a**, **b** Bar graphs depicting mRNA levels for myelin basic protein (*Mbp*; A) and proteolipid protein (*Plp1*; B) at 3 (3w) and 5 (5w) weeks of cuprizone treatment as determined by RT-qPCR analysis. *n* = 3: CC control and cuprizone 3w, CC and dLGN control and cuprizone 5w; *n* = 4: dLGN control and cuprizone 3w; *n* = 7: ON control and cuprizone 5w, *n* = 12: ON control and cuprizone 3w. **c** Scheme of a coronal mouse brain section showing OT and dLGN. The square marks the area shown in D. **d** Representative confocal images of MBP immunostained mouse brain sections at 5 weeks of cuprizone treatment (right panel) or under control conditions (left panel). Scale bar: 100 μm. **e** Bar graph depicting the density of MBP immunostaining over the area of the OT and dLGN. Two fields per slice and two slices per animal (*n* = 3: OT and dLGN control; *n* = 4: OT and dLGN cuprizone) were analyzed. **f** SBFSEM images and 3D reconstruction of a myelinated terminal branch of a retinal ganglion cell (RGC) axon within the mouse dLGN under control conditions. An RGC axon with three presynaptic boutons is pseudocolored in magenta; the last internode of myelin ensheathing this axon is pseudocolored in green. F′ shows a high magnification, unlabeled image of this axon and myelin. F″ shows a 3D reconstruction of the terminal branch of this axon and its last myelin internode. Scale bar: 1.3 μm. All bar graphs depict means ± SEMs: *p < 0.05, **p < 0.01, ***p < 0.001, not significant (ns) *p* ≥ 0.05 (one-sample *t* test; compared to set control value = 1)

To better define the extent of demyelination in areas of the visual system in the cuprizone model, we assessed the levels of messenger RNAs (mRNAs) encoding the major myelin proteins, myelin basic protein (MBP) and proteolipid protein (PLP), in the optic nerve and dLGN (Fig. 1a, b). In addition, we analyzed the corpus callosum, a white matter region most commonly studied in the cuprizone model (Fig. 1a, b). In agreement with previous reports [16], we observed reduced levels of both myelin-related mRNAs within the corpus callosum at 3 and 5 weeks of treatment, while the optic nerve remained unaffected. In addition, no changes in MBP immunoreactivity were noted within the optic tract after 5 weeks of cuprizone treatment (Fig. 1c–e). In contrast, significant reductions in *Mbp* and *Plp1* mRNA levels were observed in cuprizone-treated mice in both the dLGN (Fig. 1a, b) and the superior colliculus (not shown), another subcortical retinorecipient brain region. In addition, reduced levels of MBP immunoreactivity were seen in the dLGN (Fig. 1e). Demyelination seen in the dLGN may in part reflect changes on axons of passage, since a division of the optic tract slices through the middle of the dLGN. However, it likely also affects terminal branches of retinal axons that are normally myelinated in the dLGN (Fig. 1f). Together, these data demonstrate that demyelination occurs in visual brain centers of cuprizone-treated mice. In contrast, visual pathways, such as the optic nerve and tract, appear largely unaffected in this model.

### In the dLGN, cuprizone-induced demyelination is associated with inflammation and reactive astrogliosis

**Fig. 2:**
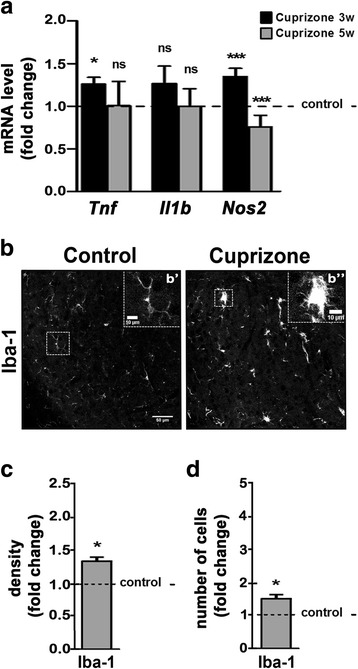
Cuprizone treatment leads to inflammatory responses within the dorsal lateral geniculate nucleus (dLGN). **a** Bar graph depicting mRNA levels for TNF-α (*Tnf*), IL-1-β (*Il1b)*, and iNOS (*Nos2*). *n* = 3: *Tnf*, *Il1b*, *Nos2* control and cuprizone 5w; *n* = 4: *Nos2* control and cuprizone 3w; *n* = 6: *Tnf* control and cuprizone 3w; *n* = 8: *Il1b* control and cuprizone 3w. **b** Representative confocal images of the dLGN within mouse brain sections immunostained for the microglia/macrophage-specific calcium-binding protein Iba-1 at 5 weeks of cuprizone treatment (right panel) or under control conditions (left panel). The insets in b’ and b” show the areas marked by the dashed white squares. Note the higher number of surveillant microglia (discrete cell bodies with thin processes, b’ under control conditions compared to the higher number of activated microglia (enlarged cell bodies with thick processes, b” upon cuprizone treatment. Scale bars: 50 and 10 μm (insets). **c**, **d** Bar graphs depicting the density of mmunostained areas (**c**) and the number of immuno-positive cells (**d**) at 5 weeks of cuprizone treatment compared to control conditions (dotted line). Two fields per slice and two slices per animal (*n* = 3: control and cuprizone) were analyzed. All bar graphs depict means ± SEMs. *p < 0.05, **p < 0.01, ***p < 0.001, not significant (ns) *p* ≥ 0.05 (one-sample *t* test; compared to set control value = 1)

To evaluate cuprizone-induced inflammation within the dLGN, the levels of mRNAs encoding inflammatory mediators and enzymes [29] were assessed (Fig. 2). In agreement with the previous observations made in the corpus callosum [30], higher mRNA levels were seen for *Tnf* (TNF-α) and *Nos2* (iNOS) but not *Il1b* (IL-1β) at 3 weeks of cuprizone treatment (Fig. 2a). At 5 weeks of cuprizone treatment, mRNA levels for *Tnf* and *Nos2* had returned to control or even below control levels (Fig. 2a), which is consistent with a resolution of pro-inflammation during the time frame of initiation of remyelination [16]. Notably, it has been shown that the microglia, next to pro-inflammatory functions, can also adopt a remyelination-promoting phenotype that is characterized by an activated morphology (amoeboid) but lacks the expression of high levels of pro-inflammatory mediators and enzymes, such as *Tnf* and *Nos2* [31]. In line with this idea, we observed, at 5 weeks of cuprizone treatment, a higher density of Iba-1 immunostaining, which was associated with an apparent amoeboid (versus ramified) microglia morphology (Fig. 2b–d).

**Fig. 3:**
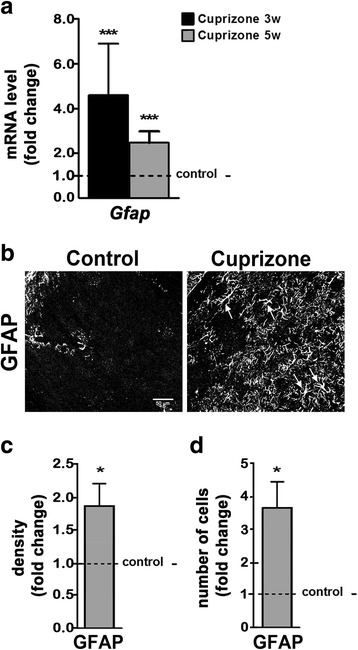
Cuprizone treatment leads to reactive gliosis within the dorsal lateral geniculate nucleus (dLGN). **a** Bar graph depicting mRNA levels of glial fibrillary acidic protein (*Gfap*) in the dLGN at 3 (3w) and 5 (5w) weeks of cuprizone treatment. *n* = 4: *Gfap* control and cuprizone 3w; *n* = 3: *Gfap* control and cuprizone 5w. **b** Representative confocal images of the dLGN within mouse brain sections immunostained for GFAP at 5 weeks of cuprizone treatment (right panel) or under control conditions (left panel). Note the presence of GFAP-positive astrocytes with hypertrophic morphology (*arrows in the right panel*). Scale bar: 50 μm. **c**, **d** Bar graphs depicting the density of immunostained areas (**c**) and the number of immuno-positive cells (**d**) at 5 weeks of cuprizone treatment compared to control conditions (dotted line). Two fields per slice and two slices per animal (*n* = 4: control; *n* = 5: cuprizone) were analyzed. All bar graphs depict means ± SEMs. *p < 0.05, **p < 0.01, ***p < 0.001, not significant (ns) *p* ≥ 0.05 (one-sample *t* test; compared to set control value = 1)

In the corpus callosum of cuprizone-treated mice, demyelination and inflammation have been found associated with reactive astrogliosis [16, 30], hallmarks of which are hypertrophy of astrocyte processes and upregulation of glial fibrillary acidic protein (*Gfap*) expression [32]. As shown in Fig. 3a, *Gfap* mRNA levels were, similar to the corpus callosum, increased in the dLGN at both 3 and 5 weeks of cuprizone treatment. Under control conditions, GFAP appeared largely absent upon immunostaining (Fig. 3b, left panel), an observation that is consistent with previous findings [33, 34]. In agreement with the observed increase in *Gfap* mRNA levels, considerably more GFAP-positive cells could be detected at 5 weeks of cuprizone treatment (Fig. 3b–d); this population of cells included GFAP-positive astrocytes displaying a hypertrophic morphology (indicated by arrows in Fig. 3b, right panel). Of note, astrogliosis, similar to microglia activation, can be associated with not only disease promoting but also regenerative processes in the CNS [35], thus supporting its presence not only during demyelination but also initial remyelination.

Taken together, the above data demonstrate that cuprizone-induced demyelination in the dLGN is associated with an inflammatory and reactive astrocyte response that is similar to the responses previously described for the corpus callosum.

### In the dLGN, cuprizone-induced inflammatory demyelination is associated with excitatory and inhibitory synaptic changes

**Fig. 4:**
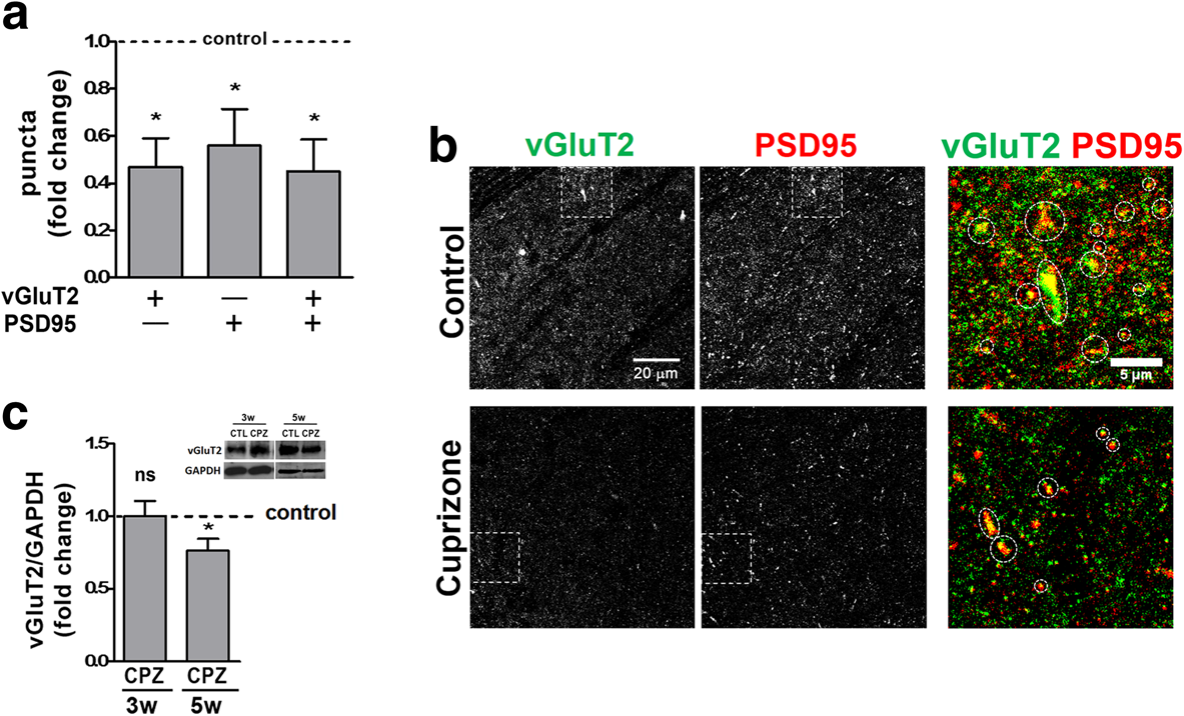
Cuprizone treatment leads to excitatory synaptic changes in the lateral geniculate nucleus. **a** Bar graph depicting the number of puncta immuno-positive for vesicular glutamate transporter 2 (vGluT2; pre-synaptic, excitatory) and postsynaptic density protein 95 (PSD95; postsynaptic) in the dLGN at 5 weeks of cuprizone treatment. Control levels were set to 1.0 (dotted line). Two fields per slice and two slices per animal (*n* = 4: control; *n* = 5: cuprizone) were analyzed. **b** Representative confocal images showing the distribution of vGluT2- and PSD95-positive synaptic densities at 5 weeks of cuprizone treatment (lower panels) or under control conditions (upper panels). The outer right panels depict the areas marked by the dotted squares; dotted circles mark vGluT2/PSD95 double-positive densities. Scale bars: 20 and 5 μm (outer right panels). **c** Bar graph depicting vGluT2 protein levels at 3 (3w) and 5 (5w) weeks of cuprizone treatment. Glyceraldehyde 3-phosphate dehydrogenase (GAPDH) protein levels were used for normalization. *n* = 4: control and cuprizone 3w; *n* = 7: control and cuprizone 5w. A representative Western blot is shown in the inset (upper right). All bar graphs depict means ± SEMs. *p < 0.05, **p < 0.01, ***p < 0.001, not significant (ns) *p* ≥ 0.05 (one-sample *t* test; compared to set control value = 1)

To investigate the effect of cuprizone-induced inflammatory demyelination on subcortical visual pathways, we evaluated potential alterations in excitatory and inhibitory synapses located within the dLGN. To assess excitatory pre-synaptic terminals from retinal ganglion cells, we examined the levels and distribution of vesicular glutamate transporter 2 (vGluT2), which is selectively present in retinal terminals within the visual thalamus [24, 36]. As shown in Fig. 4a, b, a robust decrease in the number of vGluT2-positive puncta was observed at 5 weeks of cuprizone treatment. This decrease coincided with a reduction in puncta positive for postsynaptic density protein 95 (PSD95), a marker for glutamatergic postsynaptic compartments [23]. Along with these observations, protein levels for vGluT2 were also found reduced at 5 weeks of cuprizone treatment (Fig. 4c). In contrast, no changes were observed at 3 weeks of treatment. Notably, no significant differences in the levels of *Rbfox3* mRNA were observed (not shown). *Rbfox3* mRNA encodes an alternative splicing regulator that presents the antigen recognized by NeuN antibodies, which label the nuclei of mature neurons in nearly all parts of the vertebrate nervous system [37, 38]. Thus, the decrease in excitatory synaptic contacts in the dLGN at 5 weeks of cuprizone treatment is unlikely to be a result of neuronal loss.

**Fig. 5:**
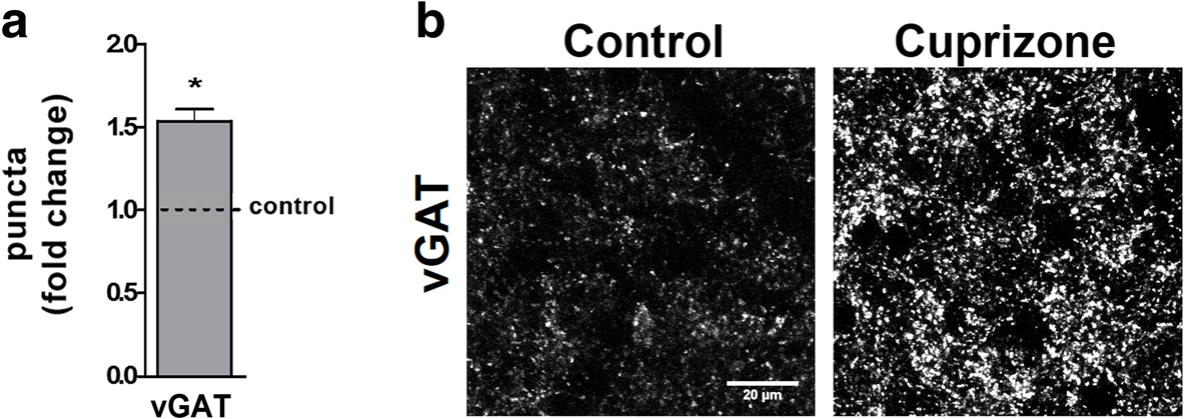
Cuprizone treatment leads to inhibitory synaptic changes in the lateral geniculate nucleus. **a** Bar graph depicting the number of puncta immuno-positive for the vesicular GABA transporter vGAT (pre-synaptic, inhibitory) in the dLGN at 5 weeks of cuprizone treatment. Control levels were set to 1.0 (dotted line). Two fields per slice and two slices per animal (*n* = 3: control and cuprizone) were analyzed. **b** Representative images showing the distribution of vGAT-positive synaptic densities at 5 weeks of cuprizone treatment (right panel) or under control conditions (left panel). Scale bar: 20 μm. The bar graph depicts mean ± SEMs. *p < 0.05, **p < 0.01, ***p < 0.001, not significant (ns) *p* ≥ 0.05 (one-sample *t* test; compared to set control value = 1)

In addition to receiving glutamatergic retinal input, thalamocortical relay cells in the dLGN also receive inhibitory inputs from a small set of local GABAergic interneurons and from GABAergic projection neurons in the thalamic reticular nucleus (TRN). To assess potential changes in the density of these inhibitory inputs into the dLGN, we performed immunohistochemistry using antibodies to the vesicular GABA transporter vGAT, which recognize all inhibitory terminals in the dLGN [24]. In contrast to the significant reduction in glutamatergic inputs into the dLGN, we observed a dramatic increase in the number of vGAT-positive puncta in the dLGN after 5 weeks of cuprizone treatment (Fig. 5).

The above data reveal robust changes in both excitatory and inhibitory inputs into the dLGN, whereby these changes appear to temporally follow the initial inflammatory demyelinating events induced by cuprizone.

### In the dLGN, cuprizone-induced inflammatory demyelination is associated with an increase in the NMDAR subunit GluN2B and an overall decrease in network activity

**Fig. 6:**
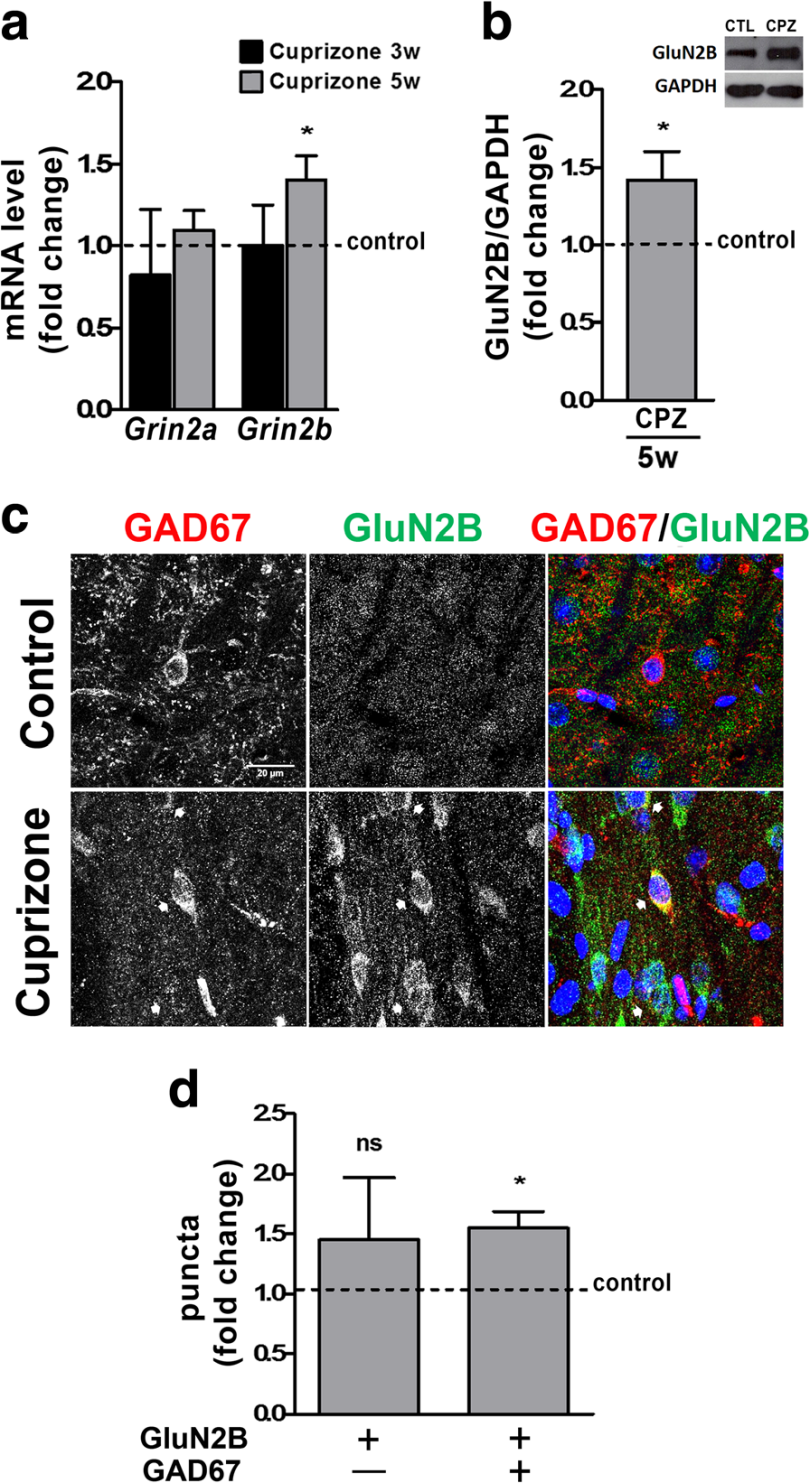
Cuprizone treatment leads to an increase in the NMDAR subunit GluN2B within the dorsal lateral geniculate nucleus (dLGN). **a** Bar graph depicting mRNA levels for NMDAR subunits GluN2A (*Grin2a*) and GluN2B (*Grin2b*) as determined by RT-qPCR analysis at 3 (3w) and 5 (5w) weeks of cuprizone treatment. *n* = 6: *Grin2a* control and cuprizone 3w and 5w; *n* = 7: *Grin2b* control and cuprizone 3w and 5w. **b** Bar graph illustrating protein levels for the NMDAR subunits GluN2B at 5 weeks of cuprizone treatment. GAPDH protein levels were used for normalization. A representative Western blot is shown in the inset (*upper right*). *n* = 5: Control (CTL) and Cuprizone (CPZ). **c** Representative confocal images depicting immunostaining for the NMDA receptor subunit GluN2B (*middle panels*) and glutamic acid decarboxylase 67 (GAD67) as a marker for inhibitory neurons (*left panels*) in the dLGN at 5 weeks of cuprizone treatment (*lower panels*) or under control conditions (*upper panels*). *Arrows in the lower panels* indicate cells labeling positive for both GAD67 and GluN2B. Scale bar: 20 μm. **d** Bar graph illustrating the number of GluN2B-positive puncta co-localizing with GAD67 at 5 weeks of cuprizone treatment. 2 fields per slice and 2 slices per animal (*n* = 4: Control; *n* = 5: Cuprizone) were analyzed. All bar graphs depict means ± SEMs. *p < 0.05, **p < 0.01, ***p < 0.001, not significant (ns) *p* ≥ 0.05 (one-sample *t* test; compared to set control value = 1)

NMDARs are voltage-dependent, hetero-multimeric ionotropic glutamate receptors that cluster on the postsynaptic membrane of excitatory synapses. Their channel properties and intracellular binding partners rely on their subunit composition, whereby a shift toward a reduced GluN2A/GluN2B ratio has been linked to a lengthening of NMDAR-mediated currents [39, 40]. Previous studies have revealed that changes in activity within the visual system are associated with changes in NMDAR subunit composition, in particular with changes in the GluN2A/GluN2B ratio [41, 42]. In light of these observations, we investigated the expression of *Grin2a* (GluN2A) and *Grin2b* (GluN2B) in the dLGN upon cuprizone treatment. As shown in Fig. 6a, both transcripts remained unchanged at 3 weeks of treatment. In contrast, *Grin2b* mRNA and GluN2B protein levels were found increased at 5 weeks of treatment (Fig. 6a, b), thus leading to an overall decrease in the GluN2A/GluN2B ratio. In the rat dLGN, it has been shown that NMDARs are present in postsynaptic membranes of synapses between retinal afferents and local interneurons [43]. Indeed, both the overall level of GluN2B expression in GAD67-positive inhibitory interneurons and the number of GluN2B-positive puncta on these interneurons were found increased in the dLGN of cuprizone-treated mice (Fig. 6c, d).

**Fig. 7:**
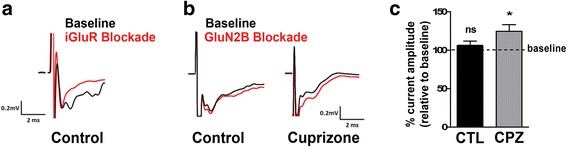
Cuprizone treatment (5 weeks) leads to a decrease in retinogeniculate network activity. **a** Representative example of traces obtained in the presence (red; iGluR blockade) or absence (black; baseline) of the glutamate receptor antagonists APV (NMDA receptor-selective) and DNQX (AMPA/kainate receptor-selective). Note that only the second peak of the traces is glutamate receptor inhibition-sensitive. **b**, **c** Representative example (**b**) and bar graph depicting the current amplitude (**c**) of traces obtained in the presence (red; GluN2B blockade) or absence (black; baseline) of Ro 25-6981, a selective blocker of NMDA receptors containing the GluN2B subunit. *n* = 5: cuprizone; *n* = 8: control. The bar graph depicts means ± SEMs. *p < 0.05, **p < 0.01, ***p < 0.001, not significant (ns) *p* ≥ 0.05 (unpaired two-tailed Student’s *t* test)

Considering the abovementioned lengthening of NMDAR-mediated currents upon decreases in the GluN2A/GluN2B ratio, the above findings point toward an increased stimulation of inhibitory interneurons and, thus, a potential overall increase in network inhibition. To assess this possibility, we performed field potential recordings during application of the GluN2B antagonist Ro25-6981 in brain slices containing both the optic tract and the dLGN [25]. Stimulation of the optic tract elicited a short latency double-peaked field negativity of which the second peak was found to be sensitive to glutamate receptor inhibition and thus identified as synaptic-dependent (Fig. 7a). Subsequent analyses were focused on this second negativity peak. As shown in Fig. 7b, c, GluN2B blockade did not change the peak amplitude in slices from control animals; however, an increase in the field negativity was observed in slices from cuprizone-treated animals. Thus, the increase in the GluN2B component was found to lead to a decrease in network activity.

Together, the above findings sustain the idea that inflammatory demyelination impacts the excitatory/inhibitory balance and can lead to an increase in inhibitory contributions to network activity in the dLGN.

## Discussion

Our findings demonstrate that in the cuprizone model demyelination occurs in retinorecipient regions of the subcortical visual system, in particular the dLGN, where it is associated with inflammatory micro- and astrogliosis. In contrast, the anterior parts of the pathway, i.e., the optic nerve and tract, appear largely unaffected in this model. These findings are consistent with previous observations [14–16, 44]. On a functional level, inflammatory demyelination within the dLGN was found accompanied by synaptic alterations leading to a shift toward retinogeniculate network inhibition. These observations are distinct from those reported for models of EAE in which inflammatory demyelination of the optic nerve [45, 46] and unbalanced synaptic hyperexcitation [3] have been reported to contribute to the overall pathology. Thus, our findings unravel a novel type of synaptic alterations accompanying inflammatory demyelination within the CNS, i.e., an excitation/inhibition balance that is shifted toward inhibitory network activity and associated with an increase in GABAergic inhibitory synapses as well as a potentially enhanced excitatory input onto inhibitory interneurons. In addition, our findings establish cuprizone-mediated inflammatory demyelination as a model for investigating pathological visual pathway changes under non-optic neuritis conditions.

Our observation that inflammatory demyelination leads to damage and/or loss of excitatory retinal inputs into the dLGN (see Fig. 4) may, at least in part, be similar to the findings made in some EAE models. In these models, synapses have been considered early targets due to their high sensitivity to even subtle inflammation-induced changes [3, 7]. Such synaptic alterations are thought to occur largely independent of axonal demyelination and neuronal cell death. Instead, they are thought to be mediated by inflammatory cytokines, in particular TNF-α, that are released from infiltrating T cells as well as activated microglia and astrocytes. In light of the increased levels of *Tnf* mRNA (see Fig. 2), a similar mechanism may drive the excitatory synapse damage/loss in the dLGN of cuprizone-treated mice. However, it is noteworthy that the reduction in the number of vGluT2-expressing synapses was observed at a stage at which pro-inflammation and *Tnf* mRNA levels began to subside. In this context, it is interesting to note that complement receptor 3-dependent synaptic engulfment by non-pathological microglia has been highlighted as a key mechanism for the elimination of ineffective synapses during development [8]. In addition, despite a lack of visible myelin breakdown and axonal degeneration in optic nerves from cuprizone-treated mice, some structural alterations and a temporal dispersion of action potentials along optic nerve axons have been reported [47]. These changes could potentially result in ineffective retinal inputs into the dLGN. It is, therefore, conceivable that microglia-mediated and complement-dependent elimination of ineffective synapses plays a role in the synaptic changes seen in the dLGN of cuprizone-treated mice. If so, such elimination would appear to uniquely affect retinal terminals in the dLGN of cuprizone-treated mice, despite the notion that microglia are capable of eliminating inhibitory synapses and have been found to do so in other injury models [48–50]. Independent of the exact mechanism, our data demonstrate that inflammatory demyelination can cause significant changes affecting the excitatory retinal inputs into the dLGN even in the absence of overt optic neuritis.

In EAE and possibly relapsing-remitting MS (see below), suppression of GABAergic tone, resulting in a stimulation of excitation, is thought to significantly contribute to synaptic loss and dysfunction. These pathophysiological changes are likely mediated by the inflammatory environment in the CNS and particularly by IL-1β release from activated microglia and infiltrating T cells. In addition, synaptic changes associated with increased levels of IL1-β have been described to include significant reductions in GluN2B localizations [51–53]. In contrast, we observed here an increase in both GluN2B and the number of vGAT-positive inhibitory interneurons, likely reflecting an increase in GABAergic tone in the dLGN of cuprizone-treated mice. Distinctive from EAE, neither T cell infiltration nor increased levels of IL-1β are considered major players in driving the pathophysiological processes in the CNS of cuprizone-treated mice [16, 30]. Thus, variations in the inflammatory demyelinating milieu may play a critical role in defining the GABAergic tone of synaptic adaptations under pathological conditions. Alternatively and/or additionally, regional heterogeneity in microglia immune alertness and responsiveness to immune stimulation has been recently reported [54]. In this context, it is worth mentioning that synaptic changes in EAE have so far only been described for CNS regions other than the dLGN. In addition, inflammatory demyelination in the EAE thalamus has been described as subtle and to occur in the absence of perivascular immune cell infiltrates typically seen in the EAE spinal cord and cerebellum [44]. Thus, regional variations in immune responses may contribute to the apparent differences in synaptic (mal)adaptions that have so far been reported for the EAE and cuprizone models.

While retinal terminals provide the primary excitatory drive onto thalamocortical relay cells in the dLGN, they comprise only a small fraction (~ 10%) of the synapses in this region [55, 56]. However, an equal (if not larger) proportion of synapses in the dLGN is GABAergic and arises from local inhibitory interneurons as well as GABAergic neurons in the nearby thalamic reticular nucleus (TRN) [57, 58]. Our data demonstrate that the dLGN of cuprizone-treated mice exhibits on the one hand reductions in the levels of VGluT2 and the numbers of excitatory retinal synapses, while at the same time the numbers of inhibitory terminals are elevated. We interpret these results to suggest a decrease in connectivity between the retina and thalamocortical relay cells that are associated with an increase in the number of inhibitory contacts onto these principle neurons. This concept is supported by our electrophysiological data (see Fig. 7). At present, it remains unclear whether the increase in vGAT-positive inhibitory nerve terminals arises from local interneurons, projection neurons in the TRN, or both. During development, impairment or loss of retinal inputs has been described to lead to exuberant and aberrant innervation by non-retinal inputs in the mouse dLGN [59, 60]. It is, therefore, conceivable that loss of excitatory drive in the visual thalamus of cuprizone-treated mice may lead to (mal)adaptive inhibitory synaptogenesis of local interneurons and/or terminal arbors arising from the TRN that is driven by an upregulation of synaptogenic signals. In the search for potential candidate molecules, BDNF has been identified as a potent inducer of inhibitory synapses that is upregulated in developing retinorecipient nuclei of enucleated rats [61–63]. In addition, BDNF has been found upregulated upon cuprizone treatment, at least in the corpus callosum where it is released by activated astrocytes [64, 65].

An interesting complexity is provided by the elevated expression levels of specifically the GluN2B NMDAR subunit in the dLGN of cuprizone-treated mice (see Fig. 6). NMDA receptors have been described to contribute to both retinogeniculate as well as corticogeniculate transmission [66, 67]. Thus, the increase seen in GluN2B-positive terminals could arise from both retinal as well cortical projections. For example, the observed increase in GluN2B-positive puncta in local GAD67-positive interneurons may reflect a compensatory mechanism in response to a reduction in excitatory retinogeniculate drive to these cells. In the hippocampus, GluN2B-containing NMDARs have been shown to positively regulate the maturation of glutamatergic input synapses in interneurons [68]. Thus, the responses seen in the dLGN of cuprizone-treated mice could reflect an attempt to induce the formation of new excitatory inputs from retinal ganglion cells in local inhibitory synapses. This idea is based on the assumption that a significant portion of the increased NMDARs resides on the postsynaptic membranes of synapses targeting local inhibitory synapses, and it is supported by our data indicating increased GluN2B protein levels within GAD67-positive dLGN interneurons (see Fig. 6). However, an alternative possibility (which is not mutually exclusive) is that a portion of the increased GluN2B subunits in cuprizone-treated animals reside on the presynaptic terminals of TRN projections into the dLGN. TRN neurons contain tonically active GluN2B-containing NMDARs on their GABAergic terminals [69]. Presynaptic NMDARs in TRN neurons facilitate GABA release and inhibition [69]. Thus, increased inhibition in the dLGN of cuprizone-treated animals may reflect elevated presynaptic NMDARs on TRN-relay cell synapses and enhanced GABA release from these terminals. As alluded to above, the synaptic changes observed under the inflammatory demyelinating conditions as they are present in the dLGN of cuprizone-treated mice may be very complex, and intricate additional studies are needed to address the mechanistic underpinnings leading to these changes. Such continuing studies would include an in vivo assessment of visual function. In this context, it is noteworthy that demyelination of the optic nerves and chiasm has been described to lead to clear alterations in both amplitude and latency of visual evoked potentials [70]. In addition, chronic cuprizone treatment was found to be associated with an impairment of visual function by recording multifocal electroretinograms [71]. Importantly, and consistent with our findings, no retinal ganglion cell degeneration was observed in this study.

From a translational point of view, complement C1q-C3-associated synaptic changes, such as those potentially contributing to the elimination of ineffective retinal inputs into the dLGN of cuprizone-treated mice, have been linked to progressive MS [72, 73]. In light of the distinct synaptic alterations seen in EAE (decrease in GABAergic tone and increase in excitation) versus cuprizone (increase in inhibitory network activity), it is also noteworthy that cuprizone-associated oligodendrocyte death [74] and an absence of significant T cell activation [75] mimic features of the pathophysiology described for primary progressive MS [76, 77]. Thus, it is tempting to speculate that the changes described here may be more representative for primary progressive MS, while those seen in EAE may be more reflective of alterations occurring in relapsing-remitting MS.

## Conclusions

Using the cuprizone model, our findings unravel a novel type of synaptic alterations accompanying inflammatory demyelination within the CNS, i.e., an excitation/inhibition balance that is shifted toward inhibitory network activity associated with an increase in GABAergic inhibitory synapses and possibly an enhanced excitatory input onto inhibitory interneurons. These findings are distinct from those describing an unbalanced increase in excitatory network activity in inflammatory demyelinating models of EAE [3]. In addition, our findings establish cuprizone-mediated inflammatory demyelination as a model for investigating pathological visual pathway changes under non-optic neuritis conditions.

## Abbreviations

CC: Corpus callosum
CNS: Central nervous system
CPZ: Cuprizone
CTL: Control
dLGN: Dorsal lateral geniculate nucleus
EAE: Experimental autoimmune encephalomyelitis
GABA: Gamma-aminobutyric acid
GAD67: Glutamate decarboxylase 1 (67 KDa isoform)
GAPDH: Glyceraldehyde 3-phosphate dehydrogenase
GFAP: Glial fibrillary protein
GluN2B: NMDAR subunit 2B
*Grin2a*: NMDAR subunit 2A
*Grin2b*: NMDAR subunit 2B
Iba-1: Microglia/macrophage-specific calcium-binding protein
*Il1b* (IL-1β): Interleukin 1 beta
MBP: Myelin basic protein
MS: Multiple sclerosis
NMDAR: *N*-Methyl-d-aspartate receptor
*Nos2* (iNOS): Inducible nitric oxide synthase
ON: Optic nerve
OT: Optic tract
PLP: Proteolipid protein
PSD95: Postsynaptic density protein 95
*Tnf* (TNF-α): Tumor necrosis factor alpha
TRN: Thalamic reticular nucleus
vGluT2: Vesicular glutamate transporter 2
